# Myotonic dystrophy type 1 accompanied with normal pressure hydrocephalus: a case report and literature review

**DOI:** 10.1186/s12883-020-01636-0

**Published:** 2020-02-12

**Authors:** Junyang Wang, Ming Liu, Wenjie Shang, Zhongqin Chen, Guoping Peng

**Affiliations:** 1 Department of Neurology, First Affiliated Hospital, Zhejiang University School of Medicine, 79 Qingchun Road, Hangzhou, 310003 China; 2 Department of Neurology, Shengzhou People’s Hospital, 666 Dangui Road, Shengzhou, 312403 China

**Keywords:** Myotonic dystrophy (DM1), Normal pressure hydrocephalus (NPH), Genetic testing, Muscular dystrophy

## Abstract

**Background:**

Myotonic dystrophy type 1 (DM1) is the most common disease that can cause muscle weakness and atrophy among adults. Normal pressure hydrocephalus (NPH) is characterized by the triad of gait disturbance, cognitive impairment and urinary incontinence. The association between DM1 and NPH is extremely rare. We report a Chinese female patient with DM1 in association with NPH.

**Case presentation:**

The patient presented with a history of 3-year of walking instability and cognitive impairment. Her brain MRI showed ventriculomegaly with normal cerebrospinal fluid (CSF) pressure and the CSF tap-test was positive, which indicated the diagnosis of probable NPH. DM1 was confirmed by genetic testing.

**Conclusions:**

Four patients with DM1-NPH association were found before. The association between NPH and DM1 may not be just a coincidence, NPH may occur in DM1 later in life and it is vital to recognize the association as a shunt surgery may improve patients’ quality of life.

## Background

Myotonic dystrophy type 1 (DM1) is the most common type of muscular dystrophy in adult. It is an autosome dominant disease which is associated with abnormal expansion of the repeated cytosine-thymine-guanine (CTG) in the 3′-untranslated regions in dystrophia myotonica-protein kinase (DMPK) gene on chromosome 19q13.3 [1]. In DM1, CTG invariably repeats for over 50 times. The disease can occur from infants to adults with the mean age of onset being in the third decade of life [2]. While the symptoms of DM1 are polymorphous [3, 4], myotonia and muscular weakness are typical clinical features, primarily affecting facial and distal limb muscles. DM1 can involve multiple systems, such as cataracts, gastrointestinal symptoms, cardiac conduction defects, hypogonadism, endocrine function impairment, and brain abnormalities [1, 5, 6]. In addition, expectation of life is markedly decreased, primarily due to aspiration pneumonia or cardiac arrhythmias [7, 8].

Normal pressure hydrocephalus (NPH) is a neurological disease characterized by the triad of gait disturbance, cognitive impairment and urinary incontinence, presenting ventriculomegaly and normal cerebrospinal fluid (CSF) pressure. It was initially described in 1965 and can be treated via ventriculoperitoneal shunt surgery [9–11]. The prevalence of probable NPH was 0.2% in people aged 70–79 years and 5.9% in those aged 80 years and older [12]. According to the American-European NPH guidelines [13], NPH can be classified into probable, possible, and unlikely categories. Probable NPH is diagnosed by hydrocephalic ventricular enlargement together with gait disturbance and either cognitive impairment or urinary incontinence.

The coexistence of myotonic dystrophy (DM) and NPH has been reported in four patients so far (Table 1) [14–16]. But none of them were confirmed by genetic testing or muscular biopsy. Here, we present a Chinese female patient with DM1 confirmed by genetic testing in association with NPH and conduct a review of the literature in order to determine clinical specific features of this co-occurrence and the possible mechanisms.

**Table 1:** Summary of five cases associating myotonic dystrophy (DM) and normal pressure hydrocephalus (NPH)

Year/Author	Gender	Onset age	Symptoms			Testing			Treatment	Improvement
			Triad of NPH	Muscle atrophy	Muscle weakness	Neuroimaging	Genetic testing	Others		
1985, Riggs	F	50	+	+	+	Obvious hydrocephalus	/	Communicating hydrocephalus on radionuclide cisternogram	Lumbar peritoneal shunt	Remarkable
1988, Christensen	F	61	+	+	+	Ventricular enlargement	/	ICP monitoring positive	Refused	/
1988, Christensen	M	75	+	+	+	Ventricular enlargement	/	ICP monitoring refused	Refused	/
2006, Delavallee	M	61	No urinary incontinence	+	+	Evans index 0.41	/	High waves on ICP monitoring	Ventriculoperitoneal shunt	80%
Present case	F	62	No urinary incontinence	+	+	Evans index 0.34	+	CSF-TT positive	Refused	/

*ICP* intracranial pressure; /, data not available; +, positive

## Case presentation

**Fig. 1:**
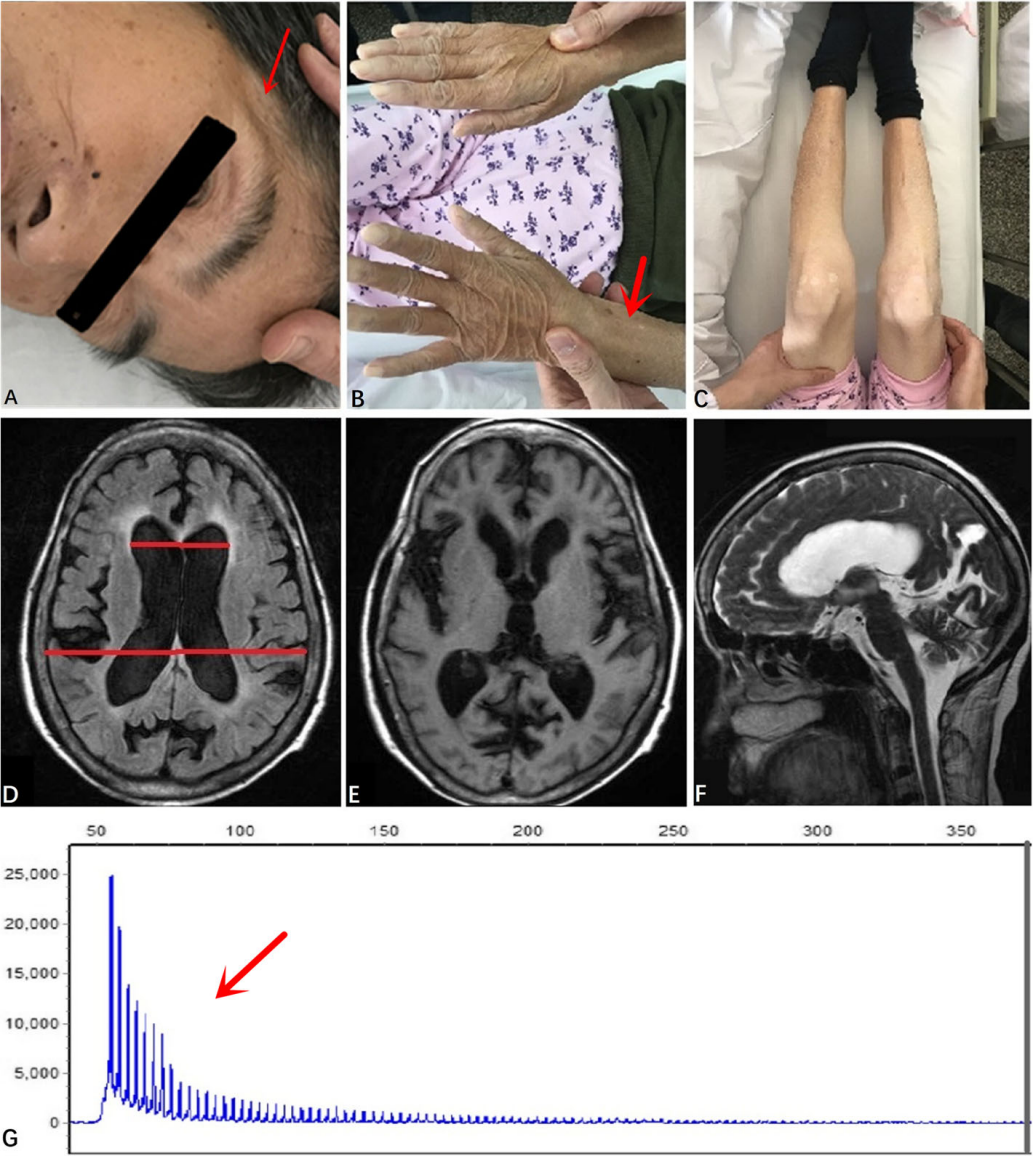
**a** Atrophy of temporalis. **b** Atrophy of bilateral distal upper limbs. **c** Atrophy of bilateral distal lower limbs. **d** Evans index = 0.34, indicating ventricular enlargement. **e**&**f** MRI scans showed atrophy on bilateral frontal, temporal and occipital lobes. **g** DNA analysis indicated that CTG repeated over 100 times

A 65-year-old Chinese woman with 1 year of grade school education came to the Department of Neurology, First Affiliated Hospital of Zhejiang University in November 2018. Her complaints included a 3-year history of walking instability which was characterized by slow walking, lower foot height, hypsokinesis, poor balance, reduced stride length, broad-based gait, and difficult starting/turning. Furthermore, she was found to have memory loss and was slow to respond. The symptoms had gradually worsened, and she was dependent on family members to help her walk. Twenty days before presenting to our clinic, she suffered thoracic vertebral compression fracture (T11–12) due to a fall at home and required surgery. The patient had no medical history of hypertension, diabetes, cardiac diseases. She had no family history of similar diseases. Her neurological examination revealed a severe gait disturbance and increased muscular tone of limbs. Marked muscle wasting was seen in the temporalis. The Medical Research Council power was 4/5 on the flexor and extensor of four limbs (Fig. 1a-c). Neuropsychological examination revealed diminished memory, prominent math difficulties and temporal-space disorientation. She scored 7/30 on the MMSE (Table 2). No abnormalities in the cranial nerves and sensory system were noted.

**Table 2:** Results of MMSE

Item	Evaluation score
Orientation to time	0/5
Orientation to place	2/5
Registration	1/3
Attention and calculation	0/5
Recall	0/3
Naming	2/2
Repetition	0/1
Reading	1/1
Writing	0/1
Complex commands	1/3
Drawing	0/1
Total	7/30

*MMSE* mini-mental state examination

Additional screens detected no abnormalities, including normal routine blood tests, biochemistry examination, coagulation function, thyroid function, tumor marker, and normal folate and vitamin B12 levels. Screens for HIV, syphilis and hepatitis were negative. Furthermore, routine CSF analysis, CSF biochemistry and CSF pressure were normal. However, first degree atrioventricular block and complete right bundle branch block was presented in the electrocardiogram (ECG), cholecystolithiasis in abdominal ultrasound, bilateral multiple thyroid nodules in the thyroid ultrasound, and chronic inflammatory foci in the lower lobes of both lungs in lung CT. Furthermore, brain MRI revealed ventriculomegaly (Evans index =0.34) (Fig. 1d) and atrophy on bilateral frontal, temporal and occipital lobes (Fig. 1e-f). Moreover, the electromyogram (EMG) indicated muscle damage with excessive myotonic discharge. Finally, genetic testing showed an over 100 CTG repeat expansion in the DMPK gene on chromosome 19q13.3, confirming the diagnosis of DM1 (Fig. 1g). Her two children also underwent genetic testing and one daughter presented with a repeat expansion on the DMPK gene.

As noted previously,the patient demonstrated gait disturbance and cognitive impairment (in absence of urinary incontinence) accompanying with normal CSF pressure and ventricular enlargement. These observations triggered a probably case of NPH [17] and cerebrospinal fluid tap-test (CSF-TT) was performed. The patient’s gait temporarily improved after CSF drainage (Table 3). A second CSF-TT was performed with a similar pattern of temporary gait improvements (Table 3). These observations are consistent with NPH, further suggesting the diagnosis of NPH. However, the patient refused shunt surgery and was discharged. A 6-month follow-up showed aggravation of symptoms and increased dependence on family members.

**Table 3:** Results of cerebrospinal fluid tap-test by using 10-m walking test

	First evaluation		Second evaluation	
	Before drainage	6 h after drainage	Before drainage	6 h after drainage
Steps	85	79 (7%↑)	75	62 (17%↑)
Time (s)	137	95 (30%↑)	68	51 (25%↑)

↑ means improvement

## Discussion and conclusions

Currently, the definite diagnosis of DM1 is made by DNA analysis. By contrast, in the past, non-molecular testing has been widely adopted which plays no role now [18] due to its inaccuracy. We searched Pubmed (index 1965–2016) for similar cases. An association between NPH and DM1 has been noted in 4 other cases, (Table 1) [14–16]. although no definitive genetic testing for DM1 was conducted in previous case studies. Here we describe a case with genetically confirmed DM1 and NPH.

Genetic testing showed that the patient had 100 CTG repeats in the DMPK gene. Consistent with a clinical presentation of DM1, the patient exhibited muscular weakness, muscle wasting of temporal and distal limb muscles. Additionally, myogenic damage and excessive myotonic discharge were noted on the EMG. For the last 3 years, she had experienced progressive gait disturbance and cognitive decline. Brain MRI showed enlarged ventricles with an Evans index of 0.34 and CSF pressure was normal. Ventricular enlargement in the presence of normal CSF pressure pointed to NPH [17], which was supported by two positive CSF-TT. NPH can be confirmed with ventriculoperitoneal shunting, unfortunately, our patient refused treatment.

DM1 brains show general atrophy and widespread gray matter volume reductions [19]. It is well established that DM1 affected central nervous system including cognitive and psychiatric dysfunction [20–30]. Deficits in several cognitive domains have been reported, including: executive function, memory, visuospatial problems, processing speed and attention [20–25].. These deficits appear to worsen as patient’s age. For instance, Sansone et al. [22] found frontal cognitive impairment worsens over time and two recent longitudinal studies have observed a progression of cognitive impairment, particularly in verbal memory, visuospatial function, attention and processing speed [31, 32]. However, the degree of decline has no correlation with the repetition number of CTG or the severity of muscular involvement [21, 31, 32]. In addition to cognitive impairments, several studies have described psychiatric disturbances in DM1 patients such as anxiety, depression, apathy, anosognosia, paranoid and aggressive traits in DM1 [20, 26–30, 33, 34]. Several psychiatric disorders such as anxiety are negatively correlated with level of education and the number of CTG repeats [30].

Cerebral ventriculomegaly is commonly reported in DM1 patients [35–37] and appears to be progressive [35]. Glantz et al. [36] reported an increased incidence (71.4%) of ventriculomegaly in DM1 patients. Neuroimaging studies using DTI and VBM have revealed extensive white and gray matter damage among DM1 patients. Brain abnormalities have been linked with the number of CTG repeats, cognitive function and muscle weakness [38–47].

The mechanism of NPH may be the obliteration of arachnoid villi (small protrusions of the arachnoid mater which can return CSF to the venous circulation), leading to the disturbances of CSF reabsorption, leading to disrupted CSF reabsorption and subsequent ventricular enlargement [48, 49]. The possible mechanism of NPH in DM1 patients may be related to widespread cell membrane defects caused by genetic abnormalities [14], leading to the arachnoid granulations [48, 50, 51]. However, the association between DM1 and NPH can be overlooked for several reasons. One might be NPH tends to occur in elderly DM1 patients, and other reasons could be that cardinal symptoms of NPH, e.g. cognitive impairment, disturbance of gait and urinary incontinence, are common in the elderly and therefore ignored as nonspecific. Mathieu and Prevost found that the mean age at death was 55.4 years for the adult-onset phenotype of DM [7]. Furthermore, according to Smulders et al. [52], the survival rate of adult-onset type of DM1 who lives to the ages 25, 45 and 65 years is 99, 88 and 18%, in comparison to the expected survival rates for unaffected adults, which are99, 95 and 78%, respectively. Therefore, the probability of adult-onset DM1 patients living to 65 years of age is significantly reduced. Our patient is now 65 and patients with NPH in association with DM1 appear to be more than 50 years old (Table 1), suggesting DM1 may result in NPH later in life.

Two of the four patients with DM1-NPH association received shunt surgery (Table 1) and were markedly improved. Although our patient refused to be treated with ventriculoperitoneal shunt, her symptoms were ameliorated after CSF drainage (Table 3), also indicating that shunt surgery is an effective therapy.

Overall, for elderly patients with DM1 presenting symptoms of gait disturbance or urinary incontinence, it is vital to consider the association of NPH. Once DM1-NPH association is considered, shunt surgery should be suggested and the patient should be transferred to neurosurgery department. In this way, it could improve the patients’ quality of life.

## Data Availability

All data generated or analysed during this study are included in this published article.

## Abbreviations

CSF: Cerebrospinal fluid
CST-TT: Cerebrospinal fluid tap-test
CTG: Cytosine-thymine-guanine
DM1: Myotonic dystrophy type 1
DMPK: Dystrophia myotonica-protein kinase
ECG: Electrocardiogram
EMG: Electromyogram
MMSE: Mini-Mental State Examination
NPH: Normal pressure hydrocephalus
